# Supplementary material for: ‘Two in One’ Cloning Vector Applied for Blunt-End and T-A Cloning with One-Step Digestion–Ligation and Screening of Positive Recombinants by Unaided Eyes
Source: Curr Issues Mol Biol. 2024 Dec 31;47(1):17. doi: 10.3390/cimb47010017 (PMC11763597; doi:10.3390/cimb47010017)
Supplement: Supplementary file 1 [file cimb-47-00017-s001.zip › cimb-3344903-supplementary.pdf]

Supplementary Figure S1.

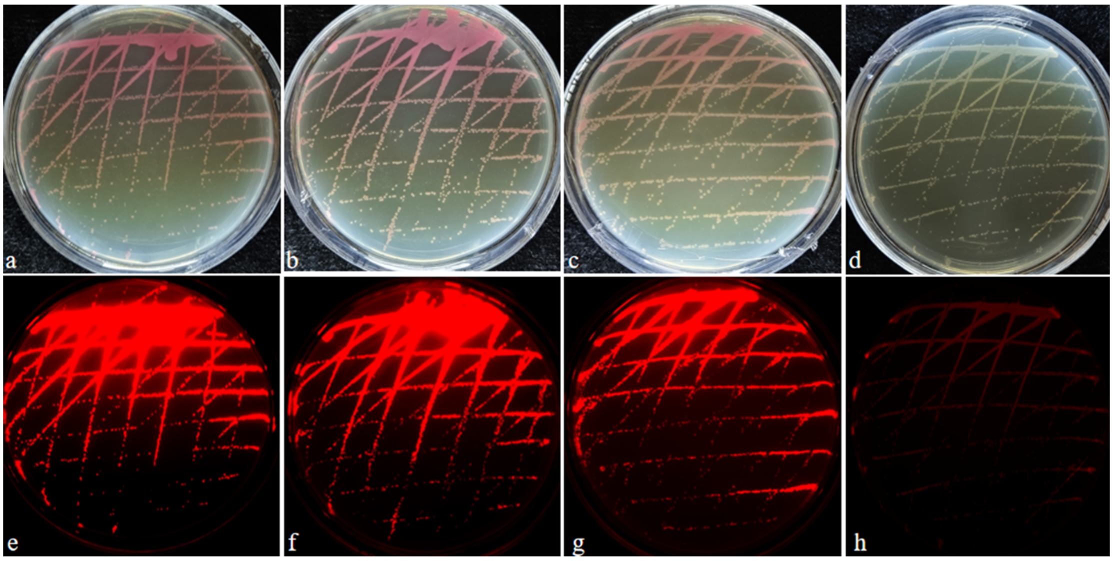

Figure S1. The effect of glucose on the expression of *mScarlet-I*of pYFRed. (a)-(d): the *E. coli* clones harboring with pYFRed under natural light; (e)-(h): the *E. coli* clones harboring with pYFRed with green excitation at 540 nm, emission at 600 nm (Tanon-5200Multi machine, Tanon Co., Ltd., China).; (a)-(e): LB medium without glucose; (b)-(f): LB medium with 0.05 mmol/L glucose; (c)-(g): LB medium with 0.1 mmol/L glucose; (d)-(h): LB medium with 0.25 mmol/L glucose.
